# A new hybrid post-translational modification—have you lost your (MARUb)les?

**DOI:** 10.1038/s44318-025-00413-4

**Published:** 2025-03-20

**Authors:** Isaac de Araujo Matos, Nícolas Carlos Hoch

**Affiliations:** Department of Biochemistry, Chemistry Institute, University of São Paulo, São Paulo, Brazil

**Keywords:** Post-translational Modifications & Proteolysis

## Abstract

New work finds ubiquitination of protein-linked mono-ADP-ribose to occur endogenously in cells upon interferon stimulation.

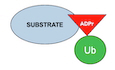

## Teaser

Recent work in *The EMBO Journal* describes a new hybrid post-translational modification of proteins, composed of a poly-ubiquitin chain that is attached to a target protein via a mono-ADP-ribose (MAR) moiety. This “MARUbylation” is likely to play important functions during interferon signalling, a core component of innate immunity.

Post-translational modifications (PTMs), such as phosphorylation, methylation, ubiquitination, and ADP-ribosylation (as well as over 300 others), regulate virtually all biological processes by altering protein function in response to a stimulus. ADP-ribosylation (ADPr) consists of the transfer of ADP-ribose units from NAD^+^ molecules onto substrate proteins or nucleic acids by enzymes of the ADP-ribosyltransferase (PARP) family, which can target many different amino acid side chains (Suskiewicz et al, 2023). Ubiquitination, which is catalyzed by the sequential action of E1, E2, and E3 ubiquitin ligase enzymes, canonically targets lysine residues via isopeptide bonds, but several additional linkage chemistries have also been identified (Dikic and Schulman, 2023).

A given protein’s enzymatic activity, cellular localization, or interaction with other proteins may be heavily influenced by a single PTM placed on a critical amino acid side chain, but oftentimes a collection of simultaneous modifications is required to integrate input from different signaling pathways, as illustrated by the “histone code”. Crosstalk between these modifications is frequent, such as the phosphorylation of a substrate protein promoting its subsequent ubiquitination and proteasomal degradation, also known as a phospho-degron. Some PTMs are polymers composed of identical monomers, such as poly-ubiquitination and poly-ADP-ribosylation, but the use of different attachment sites between these monomers (e.g., K11-, K48-, or K63-linked polyubiquitin chains), the branching of the polymeric chain (e.g., branched poly-ADPr), or the use of different chemically related monomers (e.g., glycosylation or ubiquitin/SUMO chains) can hugely increase the variety of possible polymer arrangements. Although these strategies already generate a virtually unlimited number of possible combinations, there is growing evidence for additional “hybrid PTMs”, in which chemically unrelated PTMs are added onto each other. Perhaps the best-studied example of this is the phosphorylation of ubiquitin by the PINK1 kinase in the context of mitophagy (Wauer et al, 2015).

**Figure 1:**
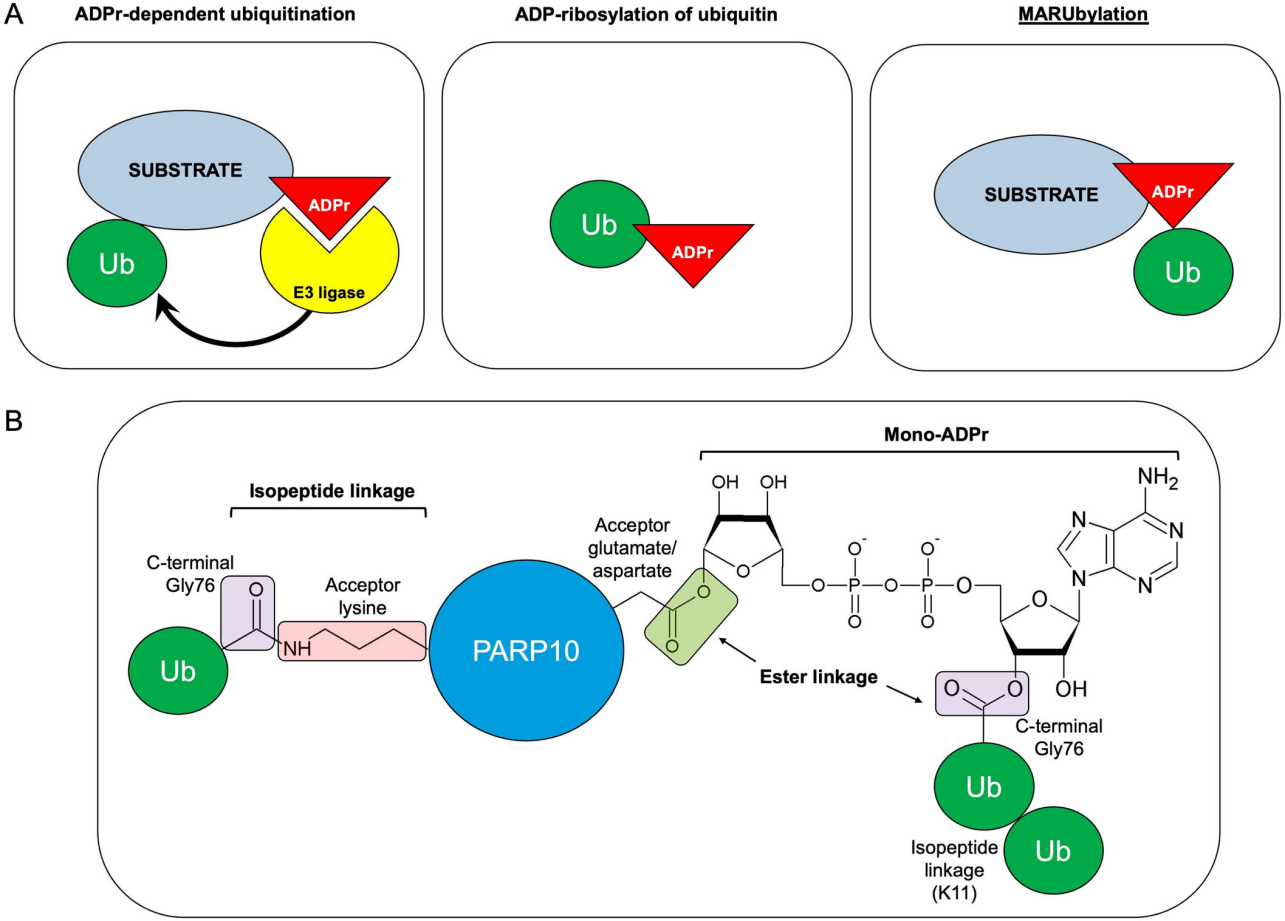
MARUbylation and other forms of ADPr-ubiquitin crosstalk. (**A**) Interplay between ADP-ribosylation and ubiquitination includes ADPr-dependent ubiquitination of a substrate, ADP-ribosylation of ubiquitin by bacterial effector proteins, and the newly discovered ubiquitination on a protein-linked mono-ADP-ribose (MARUbylation). (**B**) Occurrence of isopeptide linkages and ester linkages in substrate MARUbylation; see text for details.

In this issue of *The EMBO Journal*, Bejan et al (2025) demonstrate the formation of a new hybrid PTM composed of a protein-linked mono-ADP-ribose (MAR) that is further modified with a K11-linked polyubiquitin chain, for which the authors propose the name “MARUbylation”. These findings in human cells support and extend the recent biochemical characterization of the transfer of ubiquitin moieties onto an ADPr substrate (Zhu et al, 2022), and add to the complex interplay between ADP-ribosylation and ubiquitination, which already includes ADPr-dependent ubiquitination of a substrate (on separate amino acids) and the ADP-ribosylation of ubiquitin by bacterial effector proteins (Fig. 1A) (Zhang et al, 2011; Bhogaraju et al, 2016; Tan et al, 2024).

The authors observed that overexpression of the ADP-ribosyltransferase PARP10 in cells led to an increase in protein mono-ADP-ribosylation in a high-molecular-weight smear (Bejan et al, 2025). Curiously, this signal was sensitive to incubation with a promiscuous deubiquitinase, resulting in the collapse of the mono-ADPr smear into a single band of the expected size for the overexpressed PARP10 itself. This result indicated that PARP10 was simultaneously ubiquitinated and ADP-ribosylated, and that the ADP-ribosylation remained after the ubiquitin had been removed. Using a series of elegant chemical and enzymatic treatments of their cell lysates, they found that the ADPr moiety is linked to PARP10 via an ester linkage to glutamate/aspartate residues, and that removal or cleavage of this ADPr by several methods led to roughly 40% reduction in PARP10 ubiquitination, arguing that part of the ubiquitin was linked to the protein via ADPr. Consistent with this, the same roughly 40% of PARP10 ubiquitination was sensitive to an engineered deubiquitinase that can only cleave ester-linked ubiquitin, but not the canonical lysine isopeptide-linked ubiquitin, arguing that the ADPr-ubiquitin linkage was also via ester bonds, as already shown biochemically in a previous study (Zhu et al, 2022) (Fig. 1B).

Interestingly, the ~60% lysine-linked and the ~40% ADPr-linked ubiquitin modifications of PARP10 were both in the form of polyubiquitin chains, which the authors subjected to a collection of linkage-specific deubiquitinases to determine the polyubiquitin linkage chemistries; this led to the finding that the ADPr-linked polyubiquitin chain was almost completely formed by lysine-11 linkages between the ubiquitin units (Fig. 1B). To test if this MARUbylation is specific to PARP10, Bejan et al (2025) repeated the experiments with overexpression of another ADP-ribosyltransferase, PARP7, and found that it too is MARUbylated and that its ADPr-linked polyubiquitin chain is also K11-linked. Demonstrating that this was not an artifact of overexpression of these PARPs, the authors showed that treatment of cells with IFN-β led to an increase in PARP10 levels and the MARUbylation of two proteins with the sizes expected for both PARP10 and another ADP-ribosyltransferase, PARP14.

The finding that interferon signaling, which is a core component of innate immune responses and also shapes adaptive immunity, can induce MARUbylation is particularly exciting, as both ADP-ribosylation and ubiquitination are known to regulate interferon responses (Hoch, 2021; Heaton et al, 2016). These modifications are frequently targets of pathogen-encoded enzymes that add or remove these modifications to promote pathogenesis, often using non-canonical linkage chemistries (Dikic and Schulman, 2023; Hoch, 2021; Roberts et al, 2023). This may well be due to an evolutionary arms race between hosts and pathogens resulting in the development of ever more “eccentric” post-translational modifications, such as the MARUbylation described here, to ensure the opponent lacked appropriate mechanisms to counteract it.

Future studies will hopefully elucidate which enzymes are involved both in writing and in erasing MARUbylation, as well as identifying the target substrates of this modification, which will be critical to understand its molecular functions in cells.
